# Clinical analysis of risk factors for diarrhea associated with enteral nutrition in post-craniocerebral surgery patients

**DOI:** 10.3389/fnut.2025.1443243

**Published:** 2025-02-06

**Authors:** Lei Zhang, Lulu Zhang, Xiaojie Zhu, Leiming Xu, Lin Zhu, Hai Zhou, Shengkai Yang

**Affiliations:** Department of Neurosurgery, Binhai County People’s Hospital, Yancheng, Jiangsu Province, China

**Keywords:** diarrhea, enteral nutrition, brain injury, craniocerebral surgery, risk factor, nomogram

## Abstract

**Background:**

Patients undergoing craniocerebral surgery often require early enteral nutrition (EN) for energy support to improve neurological prognosis. However, diarrhea is a common complication associated with EN that can affect recovery and overall prognosis. This study aims to identify the clinical risk factors for diarrhea in patients receiving EN after undergoing craniocerebral surgery.

**Methods:**

The clinical data of patients hospitalized in the Department of Neurosurgery and Neurosurgical Intensive Care Unit of Binhai County People’s Hospital were retrospectively collected from January 2021 to December 2022. Variables such as sex, age, liquid preservation, infusion duration, pipeline assessment, heating during infusion, infusion rate, post-infusion rounds, and oral care were compared between diarrhea and non-diarrhea groups. Based on the variables obtained from the LASSO regression, multivariate logistic regression was employed to analyze their association with the occurrence of diarrhea. A diagnostic nomogram was constructed to predict the probability of diarrhea in patients receiving EN after craniocerebral surgery.

**Results:**

According to the inclusion and exclusion criteria, 141 patients were enrolled in this study, including 50 patients in the diarrhea group and 91 patients in the non-diarrhea group. The following factors were significantly associated with diarrhea: age ≥ 70 years (OR: 2.240; 95% CI 1.110–4.520), no pipeline assessment before EN (OR: 3.807; 95% CI 1.702–7.643), no heating of EN preparations (OR: 3.188; 95% CI 1.853–6.722), no control of normal infusion rate (OR: 1.721; 95% CI 1.136–3.890), no timely post-infusion rounds after EN (OR: 2.260; 95% CI 1.454–5.075), and no oral care during EN. Multivariate logistic regression analysis identified two independent predictors of diarrhea: no heating during EN (OR: 2.135; 95% CI 1.716–5.851) and no oral care during EN (OR: 1.125; 95% CI 1.025–1.652). A diagnostic nomogram based on these two variables was developed to predict the probability of diarrhea in postoperative craniocerebral surgery patients receiving EN. The nomogram demonstrated strong predictive performance, with an AUC of 0.848 (95% CI 0.778 to 0.918).

**Conclusion:**

Various factors contribute to the occurrence of diarrhea after receiving EN after craniocerebral surgery. A nomogram incorporating two independent predictors—lack of heating during EN infusion and absence of oral care—exhibited strong predictive ability and may serve as a useful tool for early risk assessment. These findings highlight the importance of incorporating heating protocols and maintaining oral hygiene during EN administration to reduce the risk of diarrhea and improve postoperative care outcomes.

## 1 Introduction

Cranial brain injury, caused by various factors, is a global health and socio-economic issue that can often lead to severe disability or death. Common causes of cranial brain injury include cranial trauma, cerebral hemorrhage, intracranial aneurysms, and subarachnoid hemorrhage. Patients with cranial brain injury often experience malnutrition, which is closely related to susceptibility to infections, high mortality rates, and prolonged intensive care unit (ICU) or hospital stays (1). With advancements in medicine, the mortality rate associated with cranial brain injury has been declining each year, but the average mortality rate for severe cranial brain injury remains at 30% (2). Severe cranial brain injury results in significant central nervous system damage. Fasting and hormonal changes often result in consciousness disorders, swallowing difficulties, and increased catabolism, leading to extreme physical depletion, weakened immune systems, and complications such as infections, stress ulcers, and hyperglycemia, all of which are detrimental to patient recovery. Therefore, timely, appropriate, and adequate nutritional support is vital for treating severe cranial conditions, improving nitrogen balance, enhancing immune function, and facilitating recovery (3).

Enteral nutrition (EN) plays a crucial role in providing nutritional support to neurocritical patients and has a significant influence on their prognosis (4, 5). At the same time, early EN can improve the nutritional status of patients with severe cranial brain injury and reduce complications such as intracranial and pulmonary infections. The American Society for Parenteral and Enteral Nutrition guidelines recommend initiating nutritional support as early as possible after cranial surgery (6). In cases where patients are hemodynamically stable, early EN is recommended to be started within 48 h of ICU admission to improve prognosis (1, 7). Despite these guidelines, critically ill patients with brain injury often experience malnutrition due to insufficient energy and protein intake (8).

Nutritional support is particularly important for patients undergoing cranial surgery. Patients with brain injuries mainly maintain their nutritional status through enteral nutrition (EN) and parenteral nutrition (PN), each of which has its own advantages and disadvantages. EN can regulate metabolic disorders, enhance immunity, and improve patient prognosis, aligning with the physiological needs of the human body. Therefore, early EN treatment is a significant research focus in patients with cranial injuries (1). However, patients with cranial injuries often experience early intolerance to EN (9). Studies have shown that diarrhea and gastroparesis occur in 70 and 20% of patients with severe brain injuries, respectively (10, 11). Severe diarrhea may affect nutrient absorption, leading to insufficient energy and protein intake, and may even prompt doctors to consider discontinuing EN (12).

A prospective cohort study showed that the high incidence of diarrhea after enteral nutrition (EN) in patients with traumatic brain injury is not related to EN itself but rather due to the use of antibiotics for more than a week (11). In an observational study, when the Glasgow Coma Scale (GCS) was ≤8, there was no significant difference in the incidence of stress ulcers and diarrhea between the observation group and the control group, indicating that EN does not increase the incidence of gastrointestinal complications such as stress ulcers and diarrhea. Therefore, this study aims to analyze the clinical risk factors for diarrhea in patients who received EN after cranial surgery. Additionally, it seeks to construct a nomogram to predict the likelihood of diarrhea after EN, providing guidance for clinical nutritional care of patients after cranial surgery.

## 2 Materials and methods

### 2.1 Study design

This was a retrospective observational cohort study conducted in a tertiary general hospital in China. Given the unique characteristics of patients with severe neurosurgical conditions and the design of observational studies, all studies were conducted with signed informed consent from the patient’s family. This study was approved by the Ethics Committee of Binhai County People’s Hospital (approval no. 2023-BHKYLL-025) and conducted in accordance with the ethical standards of the Declaration of Helsinki.

The clinical data of patients were retrospectively collected in the Department of Neurosurgery and Neurosurgical Intensive Care Unit of Binhai County People’s Hospital from January 2021 to December 2022. The patients were divided into diarrhea and non-diarrhea groups. In the present study, we did not perform any other intervention other than the necessary evaluation of the study.

### 2.2 Inclusion and exclusion criteria

We retrospectively collected data on 141 surgical patients who were hospitalized in the Department of Neurosurgery and Neurosurgery ICU of Binhai County People’s Hospital from January 2021 to December 2022. The inclusion criteria for this study were as follows:

Inclusion criteria: (1) computed tomography, computed tomography angiography, and magnetic resonance imaging diagnosis of hypertensive cerebral hemorrhage, intracranial aneurysm, subarachnoid hemorrhage, or acute craniocerebral injury; (2) All patients underwent surgical treatment according to the corresponding guidelines and standards for the diagnosis and treatment of the diseases (patients with hypertensive cerebral hemorrhage were treated with craniotomy and hematoma removal, patients with intracranial aneurysms were treated with aneurysm clipping, and patients with acute craniocerebral injury were treated with craniotomy and hematoma removal). (3) EN was administered 24–48 h after the operation, provided there was no evidence of fresh intracranial hemorrhage. (4) Duration of EN ≥72 h.

Exclusion criteria: (1) age > 80 or < 18 years (considering that the elderly, infants, and adolescents have different requirements for the dose and composition of enteral nutrition, these groups were not included to avoid a bias caused by age) (13, 14); (2) infections (such as *Clostridium difficile* infection); (3) patients with incomplete clinical data; (4) patients with missing follow-up information; (5) and patients with serious diseases that could affect the length of hospital stay.

### 2.3 Definition of diarrhea

Acute diarrhea is defined as loose stools or mucus occurring three or more times, and chronic diarrhea is defined as the presence of the aforementioned symptoms lasting from two weeks to one month (1). We confirmed diarrhea through observation by healthcare professionals. In this study, all diarrhea symptoms were diagnosed as acute diarrhea, and patients with chronic diarrhea were not included.

### 2.4 Mode of delivery of nutritional support

Within 24–48 h after admission, doctors, nurses, and nutritionists perform a detailed and comprehensive nutritional assessment according to the nutritional risk screening 2002 (NRS2002). In addition to some patients with stress ulcer bleeding and skull base fracture with cerebrospinal fluid leakage, the majority of patients accepted stomach tube implantation. Postoperatively, EN preparations were selected based on hospital guidelines, with initiation occurring 24–48 h after surgery, provided no fresh intracranial hemorrhage was observed. We used short peptide nutrition in the early stages of EN and gradually switched to complete protein nutrition after the patient’s gastrointestinal function and tolerance had gradually recovered. The EN suspension and emulsion solutions we used included TP, TP-HE, TPF1.5, and SP; the composition content was 500 mL/ bottle. SP is a commonly used protein-based pre-digested short peptide formula, and it is also classified as an elemental formula. The capacity density of SP is 1 kcaL/mL. The main ingredients include peptides, medium-chain triglycerides, and less carbohydrates, with no cellulose. These ingredients are more easily absorbed than other formulas and are used in the initial stages for patients with severe gastrointestinal function damage and long-term starvation. TP is a standard formula that is also a whole-protein formula and a non-elemental formula with an energy density of 1 kcaL/mL. It is used in most patients with normal intestinal function. TP-HE and TPF1.5 are energy-dense formulas with an energy density of 1.5 kcaL/mL, which reduce the water content and slightly increase the fat percentage compared to the standard formula. They are suitable for patients with limited fluid volume, such as cardiac and renal failure, and sometimes for patients with electrolyte disorders. Energy and protein requirements cannot be accurately calculated due to individual patient conditions after surgery. Therefore, patients’ energy requirements are determined through a joint assessment of the attending physician and the nutritionist. In general, patients are provided with 500 Kcal on the first day and 1,000 Kcal on the second day. In addition, intravenous protein may be added at the discretion of the physician to compensate for the lack of EN supply, and the dose may be adjusted in a timely manner according to the patient’s condition the day before. All patients had the head of the bed elevated by 30° during EN, and this position was maintained for an additional hour after the end of EN. During the EN period, the nurses regularly monitored the patients for signs of feeding intolerance. If symptoms such as chest tightness, shortness of breath, abdominal distension, and vomiting occurred, they were reported to the doctor immediately for treatment.

### 2.5 Candidate predictors

By analyzing the relevant literature on diarrhea, combining the patient’s clinical data, and conducting multidisciplinary consultation and discussion in the whole hospital, we selected the possible risk factors for diarrhea after EN in patients undergoing nutritional neurosurgery. Nine candidate predictors were identified as follows: (1) individual patient factors: sex and age; (2) nutritional support: liquid preservation, infusion duration, pipeline assessment, heating during infusion, infusion rate, post-infusion rounds, and oral care. The methods of pipeline assessment were as follows: the pipeline was evaluated every morning at 8 AM to observe whether the nasal paste on the nasogastric tube was tightly attached to the nose, whether it was loose, whether the tube was partially slipping, whether the tail was leaking fluid and air, and whether the tube was aging or cracked. Before and after EN, the catheter was rinsed with 30–50 mL of warm boiled water to keep the catheter clean and unblocked. Gastric juice was aspirated before EN, and EN was suspended if the amount of gastric retention was more than 150 mL. During continuous pumping of EN, the catheter was rinsed with 30–50 mL of warm boiled water every 4 h to keep the catheter clean and unobstructed. EN was administered using the Baitong EN pump Link-2008, equipped with a special heater and supporting pump tube that maintains a heating temperature of 40°C. Post-infusion rounds were conducted at 4–6 h intervals, and the EN rate was adjusted according to the score sheet of tolerance for enteral nutrition (2). No post-infusion rounds were performed when no assessment had been performed for more than 12 h. The patient was given a toothbrush daily to brush their teeth and tongue, a sponge brush, and 0.2% chlorhexidine solution to clean the oral mucosa (3).

### 2.6 Data collection

General information and clinical data of the patients were collected. We assessed and recorded the GCS score and NRS2002 of each patient before and after surgery. Patients underwent biochemical tests before being admitted to the hospital for surgery, and these tests were rechecked every 2 days on average after surgery. We calculated the total daily caloric and protein intake for each patient 48 h prior to EN. The nutrient solution type varied with patient conditions, and multiple solutions were often used during EN; therefore, relevant statistical data were not collected. Similarly, because neurosurgical patients are often unconscious before admission for surgery and have difficulty cooperating with weight measurements, we were not able to obtain accurate BMI measurements. Data were collected by two individuals who mutually verified the completeness, authenticity, and accuracy of the data. Complete data records were maintained by a dedicated nurse.

### 2.7 Statistical analysis

In this retrospective study, SPSS 27.0, R Studio (8.3.2), and Graph Prism 8.3.0 were used. A t-test was used to compare the measurement data, and a chi-square test, continuity correction chi-square test, or Fisher’s exact test were used to compare the count data depending on the characteristics of the data. A *p-*value of <0.05 was considered statistically significant.

Finally, a nomogram was used to predict the risk of diarrhea caused by EN in patients after craniocerebral surgery. The training set was used to identify significant predictors for high-dimensional data by LASSO regression. The optimal penalty parameter *λ* value was determined by performing 10-fold cross-validation. Then, multivariate logistic regression analysis was performed based on the variables obtained by LASSO regression, combined with univariate and multivariate logistic regression analyses of related variables affecting the occurrence of diarrhea. Finally, variables with *p*-values of less than 0.05 were included in the model, and two factors—heating during EN infusion and oral care—were selected for the final model. A nomogram was constructed. To evaluate the performance of the nomogram, the area under the receiver operating characteristic curve (AUC) was calculated for the training and validation sets. In addition, calibration curves were used to evaluate the predictive power of the nomogram.

## 3 Results

### 3.1 Patient characteristics

A total of 162 patients were enrolled in this study. Finally, 141 patients were included in this study according to the inclusion and exclusion criteria. The analysis found that there were 50 cases in the diarrhea group and 91 cases in the non-diarrhea group (Table 1). The incidence of diarrhea in neurosurgical patients receiving EN was 35.50%. Among the patients included in this study, patients aged ≥70 years were more likely to develop diarrhea than those aged <70 years (43.26% vs. 56.74%, *p* < 0.05). Patients with no pipeline assessment were more likely to have diarrhea (17.73% vs. 82.27%, *p* < 0.05). Patients receiving unheated EN fluids were more likely to develop diarrhea (52.48% vs. 47.52%, *p* < 0.05). Patients with uncontrolled infusion rates of EN preparations were more likely to have diarrhea than those with uniformly controlled infusion (31.21% vs. 68.79%, *p* < 0.05). Patients who did not have post-infusion rounds in time were more likely to have diarrhea than those who were visited in time (23.40% vs. 76.60%, *p* < 0.05). The absence of oral care during EN was associated with a higher likelihood of diarrhea (21.99% vs. 78.01%, *p* < 0.05). However, sex (female subjects 56.03% vs. male subjects 43.97%), liquid preservation (low-temperature 31.92% vs. room temperature 68.08%), and infusion duration after removal of EN (> 12 h 43.97% vs. ≤12 h 56.03%) had no statistically significant differences in the occurrence of diarrhea.

**Table 1:** Baseline demographic and clinical characteristics of diarrhea group and non-diarrhea group.

Characteristics	Total (*n* = 141)	Diarrhea (*n* = 50)	Non-diarrhea (*n* = 91)	*p* value
Sex				0.719
Male	62 (43.97%)	23 (46%)	39 (42.86%)	
Female	79 (56.03%)	27 (54%)	52 (57.14%)	
Age				**0.024**
< 70	80 (56.74%)	22 (44%)	58 (63.74%)	
≥ 70	61 (43.26%)	28 (56%)	33 (36.264%)	
Liquid preservation				0.694
Low temperatures	45 (31.92%)	17 (34%)	28 (30.77%)	
Normal temperatures	96 (68.08%)	33 (66%)	63 (69.23%)	
Infusion duration				0.727
> 12 h	62 (43.97%)	21 (42%)	41 (45.05%)	
≤ 12 h	79 (56.03%)	29 (58%)	50 (54.95%)	
Pipeline assessment				**< 0.001**
Yes	116 (82.27%)	26 (52%)	90 (98.9%)	
No	25 (17.73%)	24 (48%)	1 (1.10%)	
Heating during infusion				**< 0.001**
Yes	74 (52.48%)	9 (18%)	65 (71.43%)	
No	67 (47.52%)	41 (82%)	26 (28.57%)	
Infusion rate				**< 0.001**
Uniform velocity	97 (68.79%)	16 (32%)	81 (89.01%)	
Non-uniform velocity	44 (31.21%)	34 (68%)	10 (10.99%)	
Post-infusion rounds				**< 0.001**
Yes	108 (76.60%)	19 (38%)	89 (97.80%)	
No	33 (23.40%)	31 (62%)	2 (2.20%)	
Oral care				**< 0.001**
Yes	110 (78.01%)	20 (40%)	90 (98.90%)	
No	31 (21.99%)	30 (60%)	1 (1.10%)	

The bold values were statistically significant.

### 3.2 Univariate and multivariate logistic regression analyses of diarrhea

To identify the risk factors for diarrhea, univariate and multivariate logistic regression analyses were performed. First, univariate logistic regression analysis showed that there were six factors associated with the occurrence of diarrhea (Table 2; 95%CI 1.110–4.520).

**Table 2:** Univariate and multivariate analysis of variables related to diarrhea.

Characteristic	Univariate analysis	Multivariate analysis	
	Odds Ratio (95% CI)	*p* value	Odds Ratio (95% CI)
Sex			
Male	Reference		Reference
Female	1.136(0.570,2.270)	0.719	0.812(0.286,2.305)
Age			
< 70	Reference		Reference
≥ 70	2.240(1.110,4.520)	**0.025**	0.756(0.269,2.123)
Liquid preservation			
Low temperatures	Reference		Reference
Normal temperatures	0.863(0.414,1.799)	0.694	0.851(0.288,2.513)
Infusion duration			
> 12 h	Reference		Reference
≤ 12 h	1.130(0.564,2.274)	0.727	0.296(0.094,1.927)
Pipeline assessment			
Yes	Reference		Reference
No	3.807(1.702,7.643)	**<0.001**	1.201(0.932,2.143)
Heating during infusion			
Yes	Reference		Reference
No	3.188(1.853,6.722)	**<0.001**	**2.135(1.716,5.851)**
Infusion rate			
Uniform velocity	Reference		Reference
Non-uniform velocity	1.721(1.136,3.890)	**<0.001**	1.112(0.223,5.543)
Post-infusion rounds			
Yes	Reference		Reference
No	2.260(1.454,5.075)	**<0.001**	0.365(0.015,8.843)
Oral care			
Yes	Reference		Reference
No	1.350(1.115,1.894)	**<0.001**	**1.125(1.025,1.652)**

The bold values were statistically significant.

**Figure 1:**
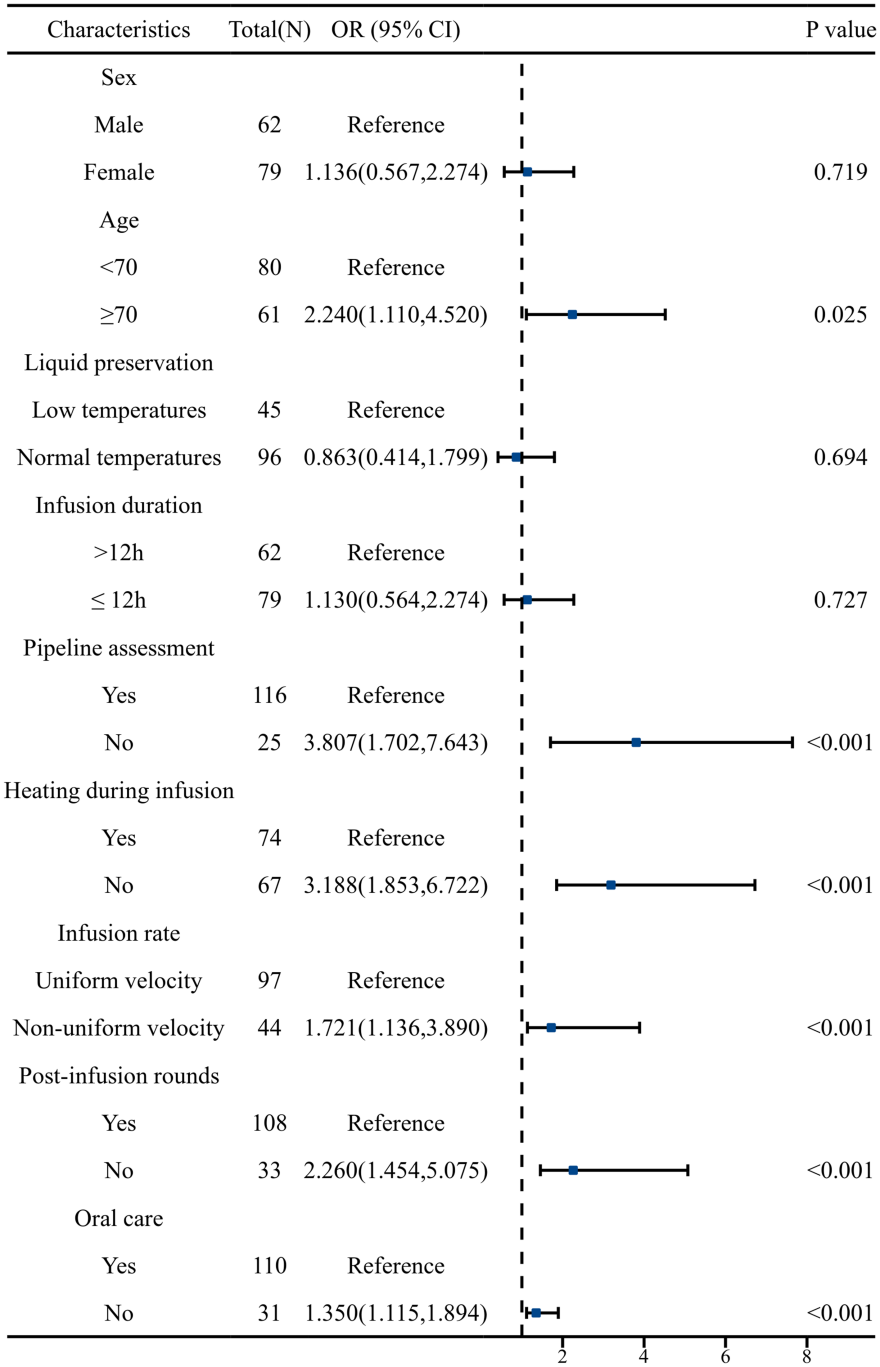
Univariate logistic regression analysis of risk factors for diarrhea caused by EN after craniocerebral surgery. OR, odds ratio.

The incidence of diarrhea was three times higher in patients who did not undergo a pipeline assessment before EN infusion compared to those who did (OR: 3.807; 95%CI 1.702–7.643). Similarly, patients receiving EN without heating the EN fluids were significantly more likely to develop diarrhea (OR: 3.188; 95%CI 1.853–6.722). The incidence of diarrhea in patients with uncontrolled normal infusion of EN preparations was almost twice that of patients with constant controlled infusion (OR: 1.721; 95%CI 1.136–3.890). The incidence of diarrhea was two times higher in patients who failed to have post-infusion rounds in time (OR: 2.260; 95%CI 1.454–5.075). The incidence of diarrhea was 1.3 times higher in patients receiving EN without oral care (OR: 1.350; 95%CI 1.115–1.894). The visualization results of the univariate logistic regression analysis are shown in Figure 1. The results of multivariate logistic regression analysis (Table 2) showed that two factors were associated with the occurrence of diarrhea, including no heating during EN infusion (OR: 2.135; 95%CI 1.716–5.851) and no oral care (OR: 1.125; 95%CI 1.025–1.652).

### 3.3 Screening for predictive factors by LASSO regression

**Figure 2:**
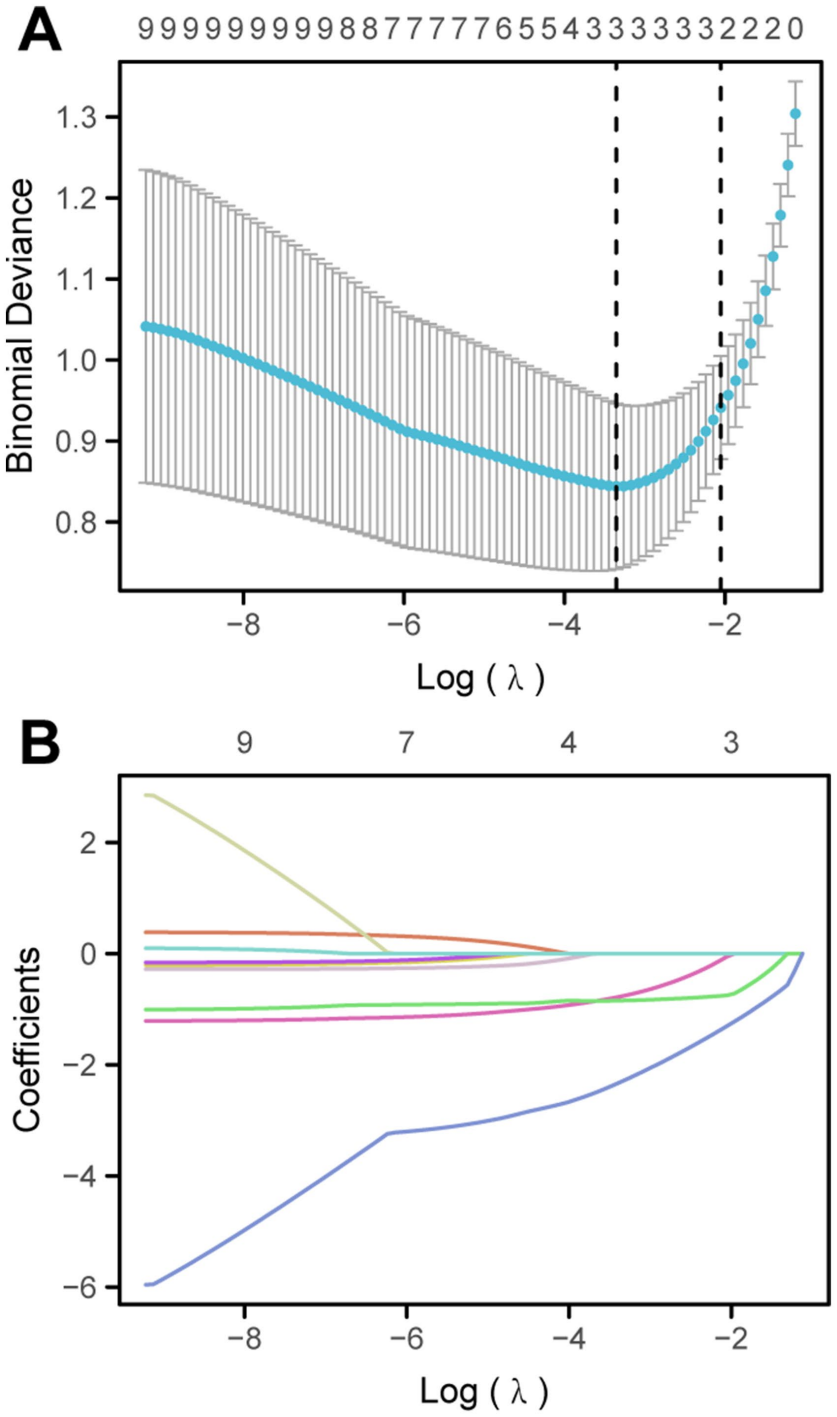
Screening for predictive factors by LASSO regression.

To more accurately identify the factors related to diarrhea, LASSO regression analysis was performed on a total of 9 variables (Figure 2). Finally, two variables were selected as risk factors for diarrhea in EN after craniocerebral surgery: no heating during EN infusion (OR: 2.135; 95%CI 1.716–5.851) and no oral care (OR: 1.125; 95% CI, 1.025 to 1.652). The results of the LASSO regression analysis show that the optimal *λ* value is λ.min = 0.028976, statistic: 0.84472, and λ.1se = 0.12838, statistic: 0.94118. To further evaluate the predictive ability of the model, we conducted the Hosmer-Lemeshow test, and the results indicate that the model has good predictive ability (*p* = 0.132).

### 3.4 Nomogram construction and validation

**Figure 3:**
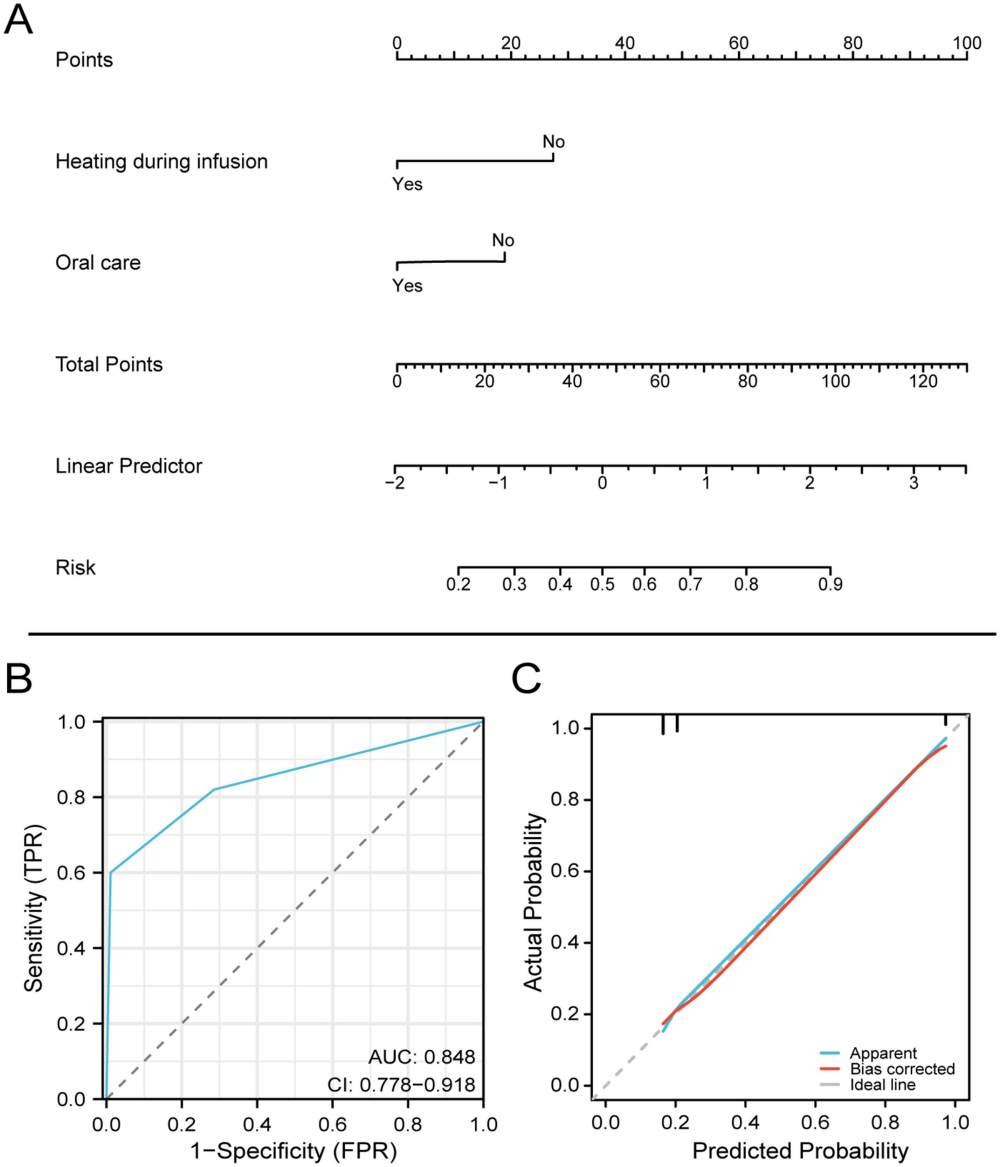
Nomogram for predicting diarrhea in patients with EN after craniocerebral surgery based on the training set.

Based on the two statistical methods of logistic regression and LASSO regression, two factors of heating and oral care during EN infusion were finally selected to construct a diagnostic nomogram for predicting the occurrence of diarrhea. Using the ROC curve verified diagnostic nomogram prediction in patient outcomes. The model AUC value is 0.848 (95% CI 0.778–0.918). Subsequently, the diagnostic calibration curve was used to evaluate the predictive probability accuracy and consistency of the diagnostic nomogram. These results show that the model has a strong predictive power, as shown in Figure 3.

## 4 Discussion

### 4.1 The need for early nutritional support

Patients with craniocerebral injury require substantial nutritional support for the following reasons. As a regulator of metabolic activity, the brain leads to a complex environment of metabolic changes following brain injury. The degree of this hypermetabolic state is proportional to the severity of injury and motor dysfunction, and the net result of these alterations is whole-body catabolism, leading to hyperglycemia, protein consumption, and increased energy requirements.

Nutrition support is necessary for patients with severe craniocerebral injury. Patients with craniocerebral trauma often suffer from severe consciousness disorders and dysphagia after surgery. This directly affects nutrient intake (4), can lead to malnutrition, and can cause various complications, including death (5). Compared to delayed EN or PN, early EN support is more conducive to improving nutritional status, reducing complications, and promoting the prognosis of patients with severe craniocerebral injury (6). Studies on the nutrition of patients with subarachnoid hemorrhage have shown that EN does not provide adequate calories and protein, and this undernutrition may be associated with higher hospital infection rates and poor outcomes (7–9). Increased caloric supply has been found to be associated with reduced mortality and in-hospital complications in patients with traumatic brain injury (10, 11).

Furthermore, in patients with traumatic brain injury, early initiation of nutritional therapy was associated with favorable outcomes, regardless of the route of administration. These findings may support the use of PN supplementation in patients with severe brain injury in whom nutritional goals cannot be achieved with EN (12). Therefore, in clinical practice, the application of nutritional support is increasingly important. Effective nutritional support can accelerate the recovery of patients and reduce complications.

### 4.2 Analysis of the incidence and influencing factors of diarrhea

In this retrospective analysis, the differences in various clinical factors between the diarrhea and non-diarrhea groups were compared after a craniocerebral operation. Univariate and multivariate analyses also showed that factors such as age ≥ 70 years, no pipeline assessment before EN infusion, no heating during infusion, no control of EN infusion rate, no timely inspection after EN infusion, and no oral care during EN were associated with the occurrence of diarrhea. Based on logistic regression and LASSO regression, two factors were selected to construct a diagnostic nomogram for predicting the occurrence of diarrhea. Heating and oral care during EN infusion were finally selected to construct a diagnostic nomogram for predicting the occurrence of diarrhea. Diagnostic nomograms suggest that lack of heating and oral care increases the risk of diarrhea.

Diarrhea is a challenging complication, often causing frustration among nursing personnel and caregivers managing EN intolerance. A total of 141 patients were included in this study. Among them, 50 patients who received EN had diarrhea, and the incidence of diarrhea was 35.50%. From a nursing perspective, this study found that patients aged≥70 years who had no pipeline evaluation before EN infusion, no heating of EN preparations, no control of normal infusion speed of EN preparations, no timely post-infusion rounds after EN infusion, and no oral care during EN were more likely to have diarrhea. In a recent multicenter prospective cohort study involving 1,109 patients with diarrhea the following independent modifiable risk factors were identified: EN (hazard ratio [RR] 1.23, 95%CI 1.16 to 1.31), days of antibiotic use (RR 1.2, 95%CI 1.02–1.03), and suppository use (RR 1.14, 95%CI 1.16–1.31; 95% CI, 1.06–1.03) (13). In contrast, opioid use was associated with a decreased incidence of diarrhea (RR 0.76, 95%CI 0.68–0.86) (13). These results were consistent although the definitions of diarrhea were different in these studies (13). *Clostridium difficile* infection should be ruled out first if a patient is treated with a course of antibiotics; however, pseudomembranous colitis is not the most common cause of diarrhea in patients receiving EN (14). Long-term antibiotic use can lead to Clostridium difficile infection, known as pseudomembranous colitis, and thus Clostridium difficile infection should be ruled out in patients who have received antibiotics, and we excluded these patients in our study.

### 4.3 Construction of a diagnostic nomogram

Nomograms are highly effective in predicting patient prognosis (15). As previously reported, our study found that heating and oral care during EN infusion had a non-negligible impact on the occurrence of EN diarrhea after craniocerebral surgery; therefore, we developed a nomogram to evaluate its ability to predict the risk ratio for the incidence of diarrhea in patients with craniocerebral surgery. Therefore, heating during infusion and oral care are predictive factors for EN tolerance in patients post-craniocerebral surgery.

### 4.4 Oral care and heating factors influencing diarrhea

Our study found that heating the EN fluid and oral care can reduce the incidence of diarrhea, which also provides a reference for EN care. The unstable temperature of nutrient infusion may increase the possibility of enteral intolerance. A nutrient solution at an appropriate temperature can promote the regular peristalsis of the intestinal smooth muscle while avoiding the influence of liquids that are too high or too low in temperature on the activities of various enzymes in the intestine (16). It has been reported that warming of the EN solution can reduce the stimulation of low temperature on the gastrointestinal tract, thereby reducing the incidence of EN intolerance such as abdominal distension and diarrhea, reducing the complications of EN, and promoting the implementation and compliance rate of EN (17, 18). Our study also confirms that the risk of diarrhea increases when EN is administered without heating. Reduced oral activity disrupts the microbial flora balance. Takeshita et al. (19) showed that the normal balance of oral microbes is disrupted, and opportunistic microbes, including *Corynebacterium*, *Peptostreptococcus*, and *Fusobacterium*, were significantly dominant in patients with tube feeding. In addition, Leibovitz et al. (20) reported a possible association between altered salivary flow, salivary biochemical composition, and increased opportunistic bacteria, including *Pseudomonas aeruginosa*, in long-term care patients with tube feeding. These changes in the oral environment may increase the risk of aspiration pneumonia. The alternative is that active oral care may improve deteriorating oral conditions in patients with tube feeding and those with restricted oral intake.

### 4.5 Strategies to improve EN tolerance

Several strategies are currently available to improve feeding tolerance in patients with brain injury. First, elevating the head of the bed by 30–45 degrees is an accepted practice and a grade I recommendation to reduce the reflux of gastric contents into the pharynx and esophagus (21). Second, enteral feeding improves feeding tolerance and reduces reflux (22). Third, although many patients after craniocerebral surgery are able to receive nearly adequate amounts of retropyloric EN early in the course of the disease, some patients may be relatively intolerant in the early stages. For these patients, we should try to gradually increase the rate of EN. Patients can generally tolerate starting EN at a rate of 20 mL/h and then advancing to a specific target of 10 to 20 mL/h every 6–8 h. Continuous EN infusion appears to be better tolerated in the early stages of neurological disease, as patients receiving bolus feeding may have higher intolerance rates than those receiving continuous feeding (23). In addition, using an enriched enteral formula (1.5 kcal/mL) or higher with a smaller capacity to provide the required quantity of heat, in many cases, will reduce the quantity of reflux or intolerance. Finally, a gastrointestinal prokinetic agent such as metoclopramide may also be considered to promote adequate peristalsis and tolerance. Prokinetic agents are not without adverse effects, so these agents should be used for a short period until the desired effect is achieved and maintained. After craniocerebral surgery, one often needs to use paralyzing agents, drugs, and other anticholinergic drugs. These drugs may cause gastrointestinal dyskinesia. Abdominal distension and postoperative ileus often inhibit patients from achieving EN goals. An aggressive bowel regimen of twice-daily liquid stool softeners and rectal laxative stimulants initiated at the beginning of EN therapy may reduce this complication (24).

### 4.6 Limitations of the research

Our study has some limitations that should be acknowledged. Due to the small sample size, there was no stratified analysis of the EN status in patients with craniocerebral injury caused by different variables, and the risk factors related to the occurrence of diarrhea with different causes were not compared. Since the same patient may receive different EN solutions depending on their condition, no further analysis was conducted to compare whether different EN programs and types would affect the incidence of diarrhea. In the future, prospective randomized controlled studies with large samples are needed to compare the incidence of diarrhea based on various factors. Finally, we selected two factors, heating and oral care during EN infusion, to construct a diagnostic nomogram to predict the occurrence of diarrhea. Further external validation is needed to evaluate the predictive ability of the model.

## 5 Conclusion

This study identified several factors associated with a greater likelihood of causing diarrhea, including age ≥ 70 years, no pipeline assessment before EN infusion, no heating during infusion, lack of control over the infusion rate, absence of timely inspection post-infusion, and no oral care during EN. According to the retrospective study, heating during infusion and oral care were identified as independent prognostic factors for survival in craniocerebral surgery patients receiving EN. These findings may provide valuable insights for improving postoperative nursing care in patients receiving EN after cerebral surgery.

## Data Availability

The original contributions presented in the study are included in the article/supplementary material. Further inquiries can be directed to the corresponding authors.
